# The effects of cannabis on mind-wandering

**DOI:** 10.1016/j.heliyon.2025.e42911

**Published:** 2025-02-21

**Authors:** Adrian Berk Safati, Wisam Almohamad Alkheder, Cassandra Justine Lowe, Daniel Smilek

**Affiliations:** a Department of Psychology, University of Waterloo, Canada; b Department of Psychology, University of Exeter, United Kingdom

**Keywords:** Attention, Mind-wandering, Sustained attention, Thought control, Executive control, Experience sampling, Cannabis

## Abstract

To examine the effects of cannabis on mind-wandering, regular cannabis smokers of legally purchased pre-rolls took part in a three-session remote study. In each 30-min session participants completed three blocks of an attention task in which they pressed the spacebar in time with a metronome tone (the Metronome Response Task), and intermittently reported their levels of spontaneous and deliberate mind-wandering. Performance on the Metronome Response Task was indexed through response time variability, with greater response variability indicating poorer performance. Following an initial ‘baseline’ block, participants were instructed to mind-wander either 20 % or 80 % of the time in the second and third blocks (counterbalanced). Critically, the first and third sessions were scheduled on days of planned abstention while the second session immediately followed the (planned) use of cannabis (an ABA design). In the baseline blocks we found that cannabis use is associated with a large increase in spontaneous mind-wandering, a smaller increase in deliberate mind-wandering, and impaired task performance. When participants were instructed to mind-wander 20 % or 80 % of the time, cannabis use reduced instruction-related changes in deliberate mind-wandering and task performance, suggesting an impairment of the regulation of mind-wandering.

## Highlights

• Examined cannabis effects on mind-wandering using a naturalistic design.
• Cannabis decreased task performance and increased mind-wandering.
• Effects of cannabis were larger for spontaneous vs. deliberate mind-wandering.
• Cannabis use diminished instruction-related changes in mind-wandering.
• Results suggest cannabis impairs the regulation of mind-wandering.

## 1 Introduction

Legalization has led to increased accessibility of retail cannabis products with higher (and riskier) potency [1,2]. There has been growing interest in the effects of cannabis use on cognitive functioning, with studies showing those under the influence of cannabis have poorer performance during everyday attention-demanding tasks (e.g. driving) [3,4] and laboratory attention tasks [[5], [6], [7]]. The psychoactive effects of cannabis are primarily attributed to Δ9-tetrahydrocannabinol (THC), a cannabinoid receptor agonist [8]. Cannabidiol (CBD), another constituent, is often of related interest, because of its reported therapeutic potential and function as an inverse agonist of the same cannabinoid receptors [9]. Here we build on previous work by exploring how cannabis use influences the ability to control one's levels of inattention, with a particular focus on the common inattentive state of mind-wandering.

Mind-wandering represents a particular form of attention lapse in which one's thoughts drift from the current task and immediate external environment to a loose internal stream of consciousness [10]. Mind-wandering experiences are of importance because they appear very common (occurring up to 50 % of the time in some contexts [11]) and are related to performance costs in a wide variety of tasks, including those examining sustained attention [[12], [13], [14]], selective attention [15,16], and divided attention [17,18]. While some have considered episodes of mind-wandering to reflect unintentional loss of control over attentional allocation [[19], [20], [21]], recent studies have shown that in many contexts, mind-wandering occurs both spontaneously (unintentionally) and deliberately (intentionally) [[22], [23], [24]]. Although correlated, these two forms of mind-wandering can be influenced by, or related to, different factors. For example, increasing participant motivation can reduce both types of mind-wandering, with a greater influence on deliberate mind-wandering than spontaneous mind-wandering in some contexts [25,26]. Likewise, rereading a passage of text can increase deliberate mind-wandering without influencing spontaneous mind-wandering [27]. As a final example, varying the difficulty of a particular attention task (i.e., the Sustained Attention to Response Task [28]) has yielded a pattern whereby levels of intentional mind-wandering were higher in the easier version than the harder version of the task, whereas unintentional mind-wandering showed the opposite pattern. These and other findings suggest that the two types of mind-wandering are governed by different cognitive mechanisms. It seems that deliberate mind-wandering is driven by “willful” processes [29] that involve directing executive control mechanisms towards explicit goals, whereas spontaneous mind-wandering is driven by automatic and reflexive mechanisms [30] that rely heavily on activating the default-mode network [31].

As the presence of deliberate mind-wandering might suggest, evidence shows that people seem able to control their level of mind-wandering [32]. Along these lines, recently, Safati et al. (2024) have shown that people can control their levels of both spontaneous and deliberate mind-wandering on demand, such that they can approximate instructed levels of mind-wandering while completing a laboratory task [33]. In these prior experiments, participants completed four blocks of the metronome response task (MRT) [[33], [34], [35]], in which they pressed the spacebar in time with a steady repeated tone. Performance on the task was indexed by response time variability, with greater variability indicating poorer performance. During the MRT, spontaneous and deliberate mind-wandering were sampled using the thought-probe technique [36]. Across blocks participants were instructed to adjust their level of mind-wandering so that in a given block they would mind-wander either 20, 40, 60, or 80 percent of the time. Using this ‘mind-wandering-on-demand’ methodology, it was found that when participants were instructed to mind-wander more they reported higher levels of mind-wandering and performed more poorly on the MRT. While deliberate (intentional) mind-wandering was more responsive to the mind-wandering instructions than spontaneous (unintentional) mind-wandering, spontaneous mind-wandering followed the same pattern, suggesting the conscious regulation of one's mental state to be more permissive or restrictive of mind-wandering effects both subtypes to different degrees. As with prior implementations of the MRT, response time variability proved to be a useful behavioural correlate for the mind-wandering reports [34,35].

There is some indication that cannabis use can influence a person's levels of mind-wandering. For example, there is indirect evidence showing disruptive effects of cannabis on the default-mode network [37,38], which involves a series of brain structures linked to mind-wandering [39]. In terms of more direct evidence, to date only two studies have documented increases in reports of mind-wandering during task performance following the oral consumption of THC [40,41]. Adam et al., had non-daily cannabis users complete a visual working memory task and intermittently sampled their experiences of whether they were “On Task”, “Mind-Wandering” or “Zoning out”, finding that the proportion of mind-wandering experiences increased when participants had ingested 15 mg of THC relative to when they were administered a placebo. Murray and Srinivasa-Desikan had infrequent cannabis users complete a working memory N-back task and retrospectively rate their levels of mind-wandering at the end of the task from 1 “not at all” to 10 “almost always”, finding dose-dependent increases in mind-wandering when participants were administered 7.5 mg and 15 mg of THC relative to a placebo. While these studies document an influence of THC on overall mind-wandering, the effects of cannabis on deliberate and spontaneous mind-wandering and the control over mind-wandering remain unknown.

Here we extend the existing literature on cannabis use and attention in several novel ways. First, we sought to separately examine the influence of cannabis use on deliberate and spontaneous episodes of mind-wandering to better characterize the role of intentionality in the effects of cannabis on mind-wandering. Second, we aimed to examine how cannabis use influences one's ability to control and regulate one's level of mind-wandering. Finally, we wanted to examine whether the relationship between mind-wandering and response variability persisted while under the influence of cannabis. We used a naturalistic ABA design, in which participants were scheduled to participate in three experimental sessions modeled after Safati et al.’s (2024) ‘mind-wandering-on-demand’ methodology. The repeated measures of the ABA design are well suited to examining the acute effects of cannabis in cannabis users, which is the primary focus of the present study. Contrasting prior work wherein participants were given controlled oral doses of THC [40,41], the present study recruited regular cannabis users who were already planning on smoking cannabis, to participate in remote sessions. Two of the sessions (Session 1 & 3; the two A sessions of the design) were on days of planned abstention from cannabis, while one of the sessions (Session 2; the B session in the design) immediately followed the (planned) smoking of cannabis. As smoking remains the most popular mode of cannabis use [42], we believe this allowed us to observe effects that generalize to cannabis users broadly.

In each session, participants first completed a ‘baseline’ block, without mind-wandering instruction, and then two ‘directed’ blocks in which they were instructed to mind-wander either 20 % or 80 % of the time (counterbalanced across participants). While the ‘baseline’ block allowed us to observe how cannabis use influences the intentionality of mind-wandering, the subsequent ‘directed’ blocks further allowed us to observe the effects of cannabis on attentional agency and the self-regulation of mind-wandering. We also asked participants general questions about their cannabis use and their levels of sleepiness during the sessions. We asked about sleepiness because cannabis can promote sleepiness [43] and because sleepiness and mind-wandering have correlated but dissociable influences on task performance [44]. This allowed us to control for sleepiness as a potential confounding factor as part of our experiential sampling during the task.

Our specific hypotheses focused on our primary aims and were based on the assumption that cannabis use would impair attentional control. Regarding our first aim, we expected that in the baseline blocks, relative to non-use, cannabis use would lead to higher rates of mind-wandering, with a greater increase in spontaneous mind-wandering relative to deliberate mind-wandering. As these cannabis-related increases in mind-wandering ought to reflect inattention to the MRT, we expected that there would be poorer performance when under the influence of cannabis than when not. Regarding our second aim, we expected that cannabis use would impair the regulation of mind-wandering in response to mind-wandering instructions such that there would be a smaller difference in mind-wandering reports between the 20 and 80 % conditions under the influence. This should be the case primarily for deliberate mind-wandering, though the effect might also bleed into spontaneous mind-wandering. Relatedly, we expected that instruction-related changes in performance would be smaller when under the influence of cannabis than when not. Finally, given that prior research using experience sampling methods with the Metronome Response Task (MRT) have found response time variability to be a behavioural correlate of mind-wandering reports [[33], [34], [35]], we expected to replicate this finding across the sessions of the present study.

## 2 Methods

### 2.1 Participants

Adults planning to smoke cannabis were recruited though posters around the University of Waterloo campus to partake in a three-session remote study. Recruitment specified individuals needed to be 1) 19–64 years of age, 2) regular cannabis smokers (2–8 times per month), 3) legally purchasing pre-rolled joints, 4) not using cannabis in combination with medications or other substances (including but not limited to tobacco and alcohol), and 5) have access to quiet distraction free spaces with video conferencing capabilities. The 2–8 times per month pattern of cannabis use was intended to help facilitate scheduling experimental sessions on days when individuals were already planning to use cannabis or abstain from cannabis use. Legal products ensured there was a standardized assessment of the cannabinoid profile that participants could report to facilitate estimates of their self-administered dose.

Based on prior studies examining the influence of cannabis use on mind-wandering and our prior studies on the topic of mind-wandering, we set a recruitment target of 50 participants. 50 adults were recruited to participate in exchange for $50 if they completed all sessions, or a pro-rated amount (i.e. $15 or $30) for partial completion of only one or two sessions. One participant was removed from the analysis due to poor compliance with task instructions, and another participant was lost to attrition. The analyzed sample (*n* = 48) consisted primarily of young adult university students, with 24 females and 23 males and 1 participant who declined questionnaires (Ages 19–53, *m* = 23.13, *SD* = 5.57).

### 2.2 Materials

#### 2.2.1 Questionnaires

At their convenience participants were asked to complete a series of questionnaires including basic demographic information (age, sex, race), the Daily Sessions, Frequency, Age of Onset, and Quantity of Cannabis Use Inventory (DFAQ-CU) [45], and the Marijuana Motives Questionnaire (MMQ) [46]. Further details and questionnaire results are available in the supplementary materials.

#### 2.2.2 Dosage survey

Cannabis products were self-selected and self-administered by participants. With their planned use of cannabis preceding Session 2 participants were asked to upload photos of the product label, the joint pre- and post-consumption, to report how “high” they felt, whether they shared the joint with others, and if so, what fraction they consumed, and to what extent they felt they “inhaled” while smoking. Product labels, photos and the fraction shared were used by researchers to estimate participants self-administered dosage.

#### 2.2.3 Metronome response task (MRT)

The MRT is a rhythmic response task that involves participants pressing the spacebar in time with a steady tone, much like a metronome [35,47]. In our study the MRT consisted of a practise block of 30 trials, followed by 3 experimental blocks, each including 354 trials (1062 trials total). The trials were each 1300 ms in duration. Each duration started with 650 ms of silence, followed by a 75-ms tone, and ended with 575 ms of silence. Participants were instructed to try to anticipate and press the spacebar in sync with the start of each tone. Performance during the MRT was assessed by computing the rhythmic response time variability (RRTv) [35], which is the variability in response times measured from the onset of the metronome tone to the participant's response, across a sequence of five trials. When computing the variance across a group of five consecutive trials, missing responses were removed to ensure that each window included five trials. For each participant and condition, variances were calculated from five trial sequences with a rolling window starting at the first trial and then shifting the window progressively through the trial sequence one trial at a time. The variances of these five-trial windows were averaged to generate the RRTv and a natural-log transformation was applied to the RRTv to increase normality of the otherwise positively skewed distribution. In the rare event that that a participant had no variability in their response time across 5 trials, a value of 0 was imputed as their transformed score.

#### 2.2.4 Experience sampling

##### 2.2.4.1 Mind-wandering thought probes

Thought probes were presented intermittently throughout the MRT. Participants were instructed to answer the thought-probe questions based on their experience before the probe either “Since the start of the block” for the first probe in each block, or “Since the last probe” for subsequent probes in the block. Probes asked, “How much were you mind-wandering …”, “Deliberately?” or “Spontaneously?”. Below each of these questions was a continuous slider anchored with “*Not at all*” on the left and “*All the time*” on the right, and participants indicated their responses by clicking and moving the sliders on the scales. The presentation order of the deliberate and spontaneous mind-wandering scales was randomized between participants. The response slider did not appear on the screen until a point on the scale was clicked to reduce bias.

##### 2.2.4.2 Karolinska Sleepiness Scale (KSS)

The Karolinska Sleepiness Scale (KSS) includes a single item asking individuals to “*Please indicate your level of sleepiness at the moment*”. The response scale ranges from 1 – “*Extremely alert*” to 10 – “*Extremely sleepy, can't keep awake*” [48].

### 2.3 Procedure

Prior to participating in the experiment individuals scheduled a 15-min information session with researchers after which they were provided with a set of questionnaires that they were asked to complete before the end of the study. The information session provided an opportunity to test remote participation capabilities and to review information and consent materials ensuring a thorough understanding of study expectations.

The information and consent process included an introduction to the concept of mind-wandering. Mind-wandering was described as a common experience in daily life when thoughts drift from one's current task and immediate external environment to an internal stream of consciousness. Participants were given common examples of situations where they might experience mind-wandering, such as when they are reading a book, going for a walk, or attending a lecture and instead of attending to their task and current environment they find their thoughts drifting to the T.V. show they watched last night, their plans for the weekend, or what it means to be happy.

A critical component of our experimental task is during specific blocks participants are directed to mind-wander different amounts. To ensure clarity in how we expected participants to respond to these instructions participants were given examples of mentation that we do not consider to be mind-wandering such as performing mental arithmetic or counting objects in their environment. While counting and arithmetic may reflect attentional disengagement, they are best characterized as alternative tasks and lack the movement of thoughts that typically define mind-wandering [39,49].

To help distinguish between the subtypes of spontaneous and deliberate mind-wandering, we used the analogy of a university lecture. We explained that spontaneous mind-wandering was akin to the experience of sitting in a class and trying to focus but finding that despite their intention to be attentive to the lecture their thoughts start drifting away unintentionally from the lecture content to lecture-unrelated thoughts. We explained that with deliberate mind-wandering instead of trying to attend to the lecture, one might wilfully try to tune out the speaker to let their mind-wander elsewhere. When instructed to mind-wander the participants were told they could engage in either deliberate or spontaneous mind-wandering, and when asked to report on their experiences, there could be some overlap between the two.

Participants then completed three 30-min sessions that primarily included the Metronome Response Task (MRT). The first and third sessions were scheduled on days of planned abstention while the second session immediately followed the planned use of cannabis (an ABA design). Participants were encouraged to complete all three sessions within a week and at similar times of day. Researchers provided maximal flexibility in scheduling and rescheduling to align with participant schedules. Participants were informed there would be no penalty for non-compliance with planned cannabis use, and participation in sessions would be permitted with or without the use of cannabis.

The experimental sessions were conducted remotely over Microsoft Teams or Zoom by participant preference. Participants were encouraged to find quite spaces free from distraction. Researchers turned their microphone and camera off between instruction delivery. The remote experimental sessions allowed 1) participants to compete the experiments in a familiar environment, 2) researchers to provide clarity in task instructions, and 3) researchers to supervise participant engagement in experimental tasks.

In each experimental session participants completed a short practise block followed by a ‘baseline’ block and two ‘directed’ blocks of the MRT while intermittently reporting on their levels of spontaneous and deliberate mind-wandering. For the ‘baseline’ block, participants were not given any instructions about a desired level of mind-wandering. Following this, during the ‘directed’ blocks participants were instructed to mind-wander either 20 % or 80 % of the time (counterbalanced). The MRT blocks were intermittently interrupted with probes asking participants to report on their levels of deliberate and spontaneous mind-wandering on a single screen. In the practise block mind-wandering probes appeared after the 10th and 20th trial, in the experimental blocks mind-wandering probes appeared after every 59 trials. Each block began and ended with the Karolinska Sleepiness Scale (KSS). The task was presented using version 7.3 of jsPsych [50].

## 3 Analysis

The dependent measures included reports of spontaneous and deliberate mind-wandering (Models 1.1, 2.1, 3.1), and RT variance (Models 1.2, 2.2). Herein we collapsed the two sober sessions in our analysis when comparing as a function of cannabis use, an analysis of all the sessions separately is available in the supplementary materials. These outcomes were analyzed using the lme4 package [51] in R version 4.3.3 [52] with linear mixed effects models. Post-hoc analyses were performed using the emmeans package with Tukey's HSD to adjust for multiple pairwise comparisons [53]. The mixed effects model outcomes are presented as ANOVA results in Table 1, Table 2, Table 3; the ANOVAs assessed significance using the Satterthwaite approximation for degrees of freedom with the lmerTest package [54]. One participant experienced a minor unplanned interruption during their third session, and the corresponding portion of their data was excluded from analysis.

**Table 1:** ANOVA results for Linear Mixed Effects Models Examining Baseline Blocks.

Model	Parameter	*SS*	*MS*	*df*_*Num*_	*df*_*Den*_	*F*	*p*	*η*^*2*^
**Model 1.1**	Model Specification: MW ∼ Cannabis*Type + KSS + (1 | ID:Type) Model Data: Baseline Blocks, Observations = 1728 Performance: *R*^2^_Marginal_ = 0.213, *R*^2^_Conditional_ = 0.609							

	Cannabis	44561.52	44561.52	1	1636.9	137.135	<0.001	0.08
	Type	12850.20	12850.20	1	95.2	39.546	<0.001	0.29
	KSS	12610.67	12610.67	1	1722.6	38.809	<0.001	0.02
	Cannabis:Type	15027.55	15027.55	1	1629.1	46.246	<0.001	0.03

**Model 1.2**	Model Specification: LN_RTv ∼ Cannabis + KSS + (1 | ID) Model Data: All Blocks, Observations = 50179 Performance: *R*^2^_Marginal_ = 0.006, *R*^2^_Conditional_ = 0.212							

	Cannabis	367.42	367.42	1	50146.1	309.111	<0.001	<0.01
	KSS	8.88	8.88	1	49759.1	7.467	0.006	<0.01

Predictor significance was assessed using the Satterthwaite approximation for degrees of freedom.

**Table 2:** ANOVA results for Linear Mixed Effects Models Examining Directed Blocks.

Model	Parameter	*SS*	*MS*	*df*_*Num*_	*df*_*Den*_	*F*	*p*	*η*^*2*^
**Model 2.1**	Model Specification: MW ∼ Instruction*Cannabis*Type + KSS + (Instruction | ID:Type) Model Data: Instructed Blocks, Observations = 3454 Performance: *R*^2^_Marginal_ = 0.234, *R*^2^_Conditional_ = 0.579							

	Instruction	75039.33	75039.33	1	97.6	178.503	<0.001	0.65
	Cannabis	24437.54	24437.54	1	3266.2	58.132	<0.001	0.02
	Type	4441.4	4441.4	1	94.7	10.565	0.002	0.10
	KSS	22110.99	22110.99	1	3129.3	52.598	<0.001	0.02
	Instruction:Cannabis	6427.83	6427.83	1	3257.6	15.291	<0.001	<0.01
	Instruction:Type	11463.4	11463.4	1	97.6	27.269	<0.001	0.22
	Cannabis:Type	46907.77	46907.77	1	3256.3	111.584	<0.001	0.03
	Instruction:Cannabis:Type	4663.89	4663.89	1	3256.3	11.094	0.001	<0.01

**Model 2.1a**	Model Specification: Deliberate ∼ Instruction*Cannabis + KSS + (Instruction | ID) Model Data: Instructed Blocks, Observations = 1727 Performance: *R*^2^_Marginal_ = 0.291, *R*^2^_Conditional_ = 0.597							

	Instruction	60420.68	60420.68	1	48.5	145.161	<0.001	0.75
	Cannabis	1048.3	1048.3	1	1633.6	2.519	0.113	<0.01
	KSS	990.13	990.13	1	1400.9	2.379	0.123	<0.01
	Instruction:Cannabis	10352.55	10352.55	1	1628.5	24.872	<0.001	0.02

**Model 2.1b**	Model Specification: Spontaneous ∼ Instruction*Cannabis + KSS + (Instruction | ID) Model Data: Instructed Blocks, Observations = 1727 Performance: *R*^2^_Marginal_ = 0.158, *R*^2^_Conditional_ = 0.542							

	Instruction	17529.77	17529.77	1	49.2	41.833	<0.001	0.46
	Cannabis	64167.56	64167.56	1	1632.3	153.131	<0.001	0.09
	KSS	31999.49	31999.49	1	1641	76.364	<0.001	0.04
	Instruction:Cannabis	136.65	136.65	1	1628.3	0.326	0.568	<0.01

**Model 2.2**	Model Specification: LN_RTv ∼ Instruction*Cannabis + KSS + (Instruction | ID) Model Data: All Blocks, Observations = 100240 Performance: *R*^2^_Marginal_ = 0.014, *R*^2^_Conditional_ = 0.266							

	Instruction	5.76	5.76	1	47.5	4.359	0.042	0.08
	Cannabis	493.65	493.65	1	100173.5	373.665	<0.001	<0.01
	KSS	603.4	603.4	1	89156	456.737	<0.001	<0.01
	Instruction:Cannabis	32.14	32.14	1	100143.3	24.327	<0.001	<0.01

Predictor significance was assessed using the Satterthwaite approximation for degrees of freedom.

**Table 3:** ANOVA table for the linear mixed effects model examining relationship between mind-wandering reports and preceding task performance.

Model	Parameter	*SS*	*MS*	*df*_*Num*_	*df*_*Den*_	*F*	*p*	*η*^*2*^
**Model 3.1**	Model Specification: MW ∼ PrecedingRTv*Type*Session + (1 | ID:Type) Model Data: Instructed Blocks, Observations = 5182 Performance: *R*^2^_Marginal_ = 0.120, *R*^2^_Conditional_ = 0.407							

	PrecedingRTv	90232.439	90232.439	1	4819.432	152.289	<0.001	0.03
	Type	9385.822	9385.822	1	2794.509	15.841	<0.001	0.01
	Session	8097.233	4048.617	2	5092.484	6.833	0.001	<0.01
	PrecedingRTv:Type	5262.547	5262.547	1	4819.432	8.882	0.003	<0.01
	PrecedingRTv:Session	4739.277	2369.639	2	5092.823	3.999	0.018	<0.01
	Type:Session	1510.029	755.014	2	5092.484	1.274	0.280	<0.01
	PrecedingRTv:Type:Session	4038.795	2019.398	2	5092.823	3.408	0.033	<0.01

Predictor significance was assessed using the Satterthwaite approximation for degrees of freedom.

Models 1.1 and 1.2 examine the data from the ‘baseline’ (non-instructed) block, Models 2.1 and 2.2 examine the data from the ‘directed’ blocks, Model 3.1 examines data from all blocks. In Models 1.1–2.2 average sleepiness scores from the KSS administered at the beginning and end of each block were included in the fixed effects to account for sleepiness as a potential confounding factor. An analysis of the KSS as dependent measure is available in the supplementary materials. In Models 1.1, 2.1, and 3.1 we included separate intercepts for spontaneous and deliberate mind-wandering reports as these subtypes are distinct constructs. Models 1.2 and 2.2 included random slopes for the effect of instructions to better account for individual differences in the response to the instructions to mind-wander 20 % or 80 % of the time. Post-hoc analysis of the models using the SIMR package in R [55], confirms convergence without errors across 100 simulations, supporting that the analysis was appropriately powered. The code to run this analysis has been made available as part of our OSF repository.

## 4 Results

Participant reports and dosage survey results indicated full compliance with the planned sessional abstinence and use of cannabis. During the cannabis sessions we estimate participants self-administered an average dose of 83.45 mg ± 47.24 (3.01–246.12) of THC, and 0.52 mg ± 1.31 (0–8.50) of CBD.

### 4.1 The influence of cannabis during baseline

**Fig. 1:**
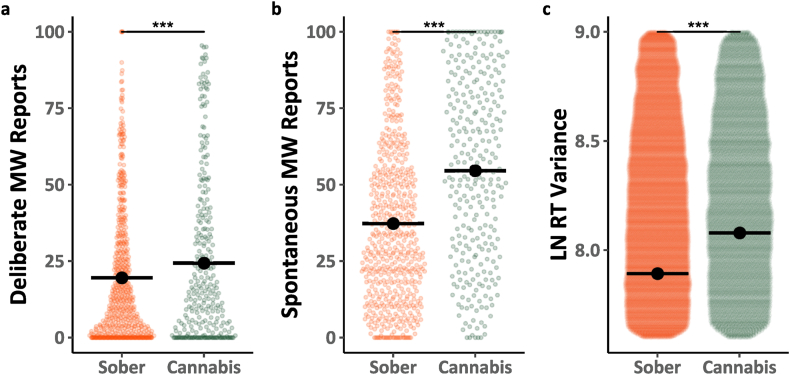
Mind-wandering reports and MRT performance during baseline blocks. The coloured points illustrate raw participant responses, with the y-axis for Panel c) restricted to better highlight mean differences. The estimated marginal means from Model 1.1 for Panels a) & b), Model 1.2 for Panel c) are presented in black. Pairwise comparisons with significance scores are shown at the top, Tukey's HSD was used to adjust for multiple comparisons. The rhythmic response time variability scores presented in Panel c) was transformed by applying a natural logarithm function. (∗ = p < 0.05, ∗∗ = p < 0.01, ∗∗∗ = p < 0.001).

We began by analyzing data from the baseline block, the first block in each session in which participants were not given any instructions to mind-wander. Fig. 1a–b shows participant mind-wandering responses in the baseline block as a function of type of report (Deliberate, Spontaneous) and cannabis use (Sober, Cannabis) with the data for the two sober sessions (Sessions 1 and 3) combined and compared to data from the cannabis session (Session 2). Analyses of the data (see Model 1.1; Table 1) revealed several important findings. First, there was a main effect of mind-wandering type *F*(1, 95.2) = 39.546, *p* < .001, *η*^2^ = 0.29, such that overall people reported more spontaneous than deliberate mind-wandering. Second, there was a main effect of cannabis use *F*(1, 1636.9) = 137.135, *p* < .001, *η*^2^ = 0.08, whereby overall mind-wandering was greater when participants were under the influence of cannabis then when they were sober. Third, there was an interaction *F*(1, 1629.1) = 46.246, *p* < .001, *η*^2^ = 0.03, whereby the effect of cannabis use was much more prominent for spontaneous than for deliberate mind-wandering. Fig. 1c includes the MRT RT variability in the baseline block as a function of cannabis use. Analyses of these data (Model 1.2; Table 1) revealed a main effect of cannabis *F*(1, 50146.1) = 309.111, *p* < .001, *η*^2^ = 0.01, whereby performance was poorer (RT variance was higher) right after participants smoked cannabis then sessions when they were sober.

### 4.2 The influence of cannabis on directed mind-wandering

**Fig. 2:**
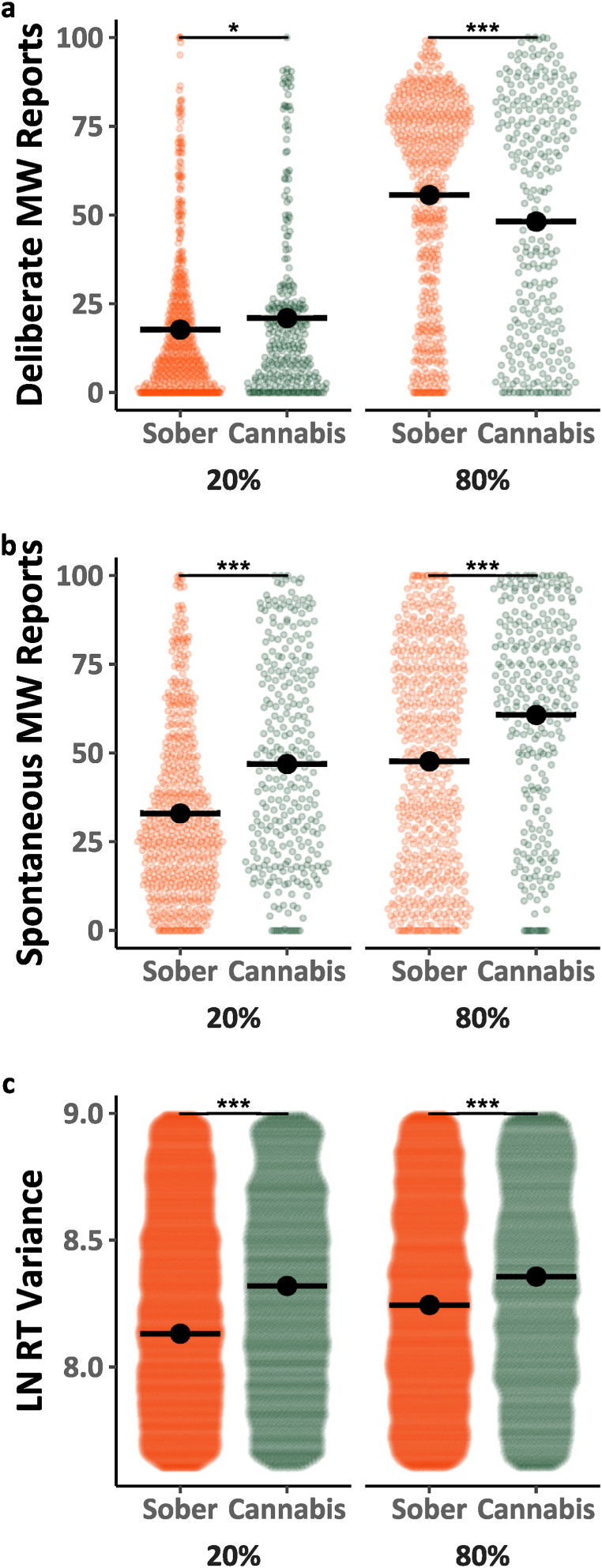
Mind-wandering reports and MRT performance during directed blocks. The coloured points illustrate raw participant responses, with the y-axis for Panel c) restricted to better highlight mean differences. The estimated marginal means from Model 2.1 for Panels a) and b), and Model 2.2 for Panel c), are presented in black. Pairwise comparisons with significance scores are shown at the top, Tukey's HSD was used to adjust for multiple comparisons. The rhythmic response time variability scores presented in Panel c) were transformed by applying a natural logarithm function. (∗ = p < 0.05, ∗∗ = p < 0.01, ∗∗∗ = p < .001).

Next, we analyzed data from the two directed mind-wandering blocks (20 % and 80 % instructed mind-wandering) to examine the effects of cannabis use on people's ability to control their levels of mind-wandering to match instructed amounts. Mind-wandering reports in the directed blocks are depicted in Fig. 2a–b as a function of mind-wandering type (Deliberate, Spontaneous), cannabis use (Sober, Cannabis) and instruction (20 %, 80 %). Analyses of the mind-wandering reports (Model 2.1, Table 2) revealed that all the main effects and interactions were statistically significant *F*(1, 3256.3) = 11.094, *p* = .001, *η*^2^ < 0.01. Before decomposing the three-way interaction, it is worth noting that Model 2.1 demonstrates replication of several prior findings. Specifically, there was a significant increase in mind-wandering from the 20 %–80 % instruction blocks *F*(1, 97.6) = 178.503, *p* < .001, *η*^2^ = 0.65 (main effect of instructions), indicating that instructions were effective, as well as a larger impact of instructions on deliberate than spontaneous mind-wandering *F*(1, 97.6) = 27.269, *p* < .001, *η*^2^ = 0.22 (the instruction by type interaction). To decompose the three-way interaction, we analyzed the deliberate and spontaneous mind-wandering reports separately. Analysis of deliberate mind-wandering reports (see Model 2.1a; Table 2) revealed a significant instruction by session interaction *F*(1, 1628.5) = 24.872, *p* < .001, *η*^2^ = 0.02. This interaction was driven by a smaller effect of instructions when participants were under the influence of cannabis, than when they were not. In contrast, a comparable analysis of spontaneous mind-wandering reports (Model 2.1b; Table 2) showed that cannabis use, and the mind-wandering instructions had independent effects on these reports, as the instruction by cannabis interaction did not reach significance *F*(1, 1628.3) = 0.326, *p* < .001, *η*^2^ < 0.01.

The MRT RT variability data from the instructed blocks are shown in Fig. 2c. They were analyzed (see Model 2.2; Table 2) as a function of cannabis use (Sober, Cannabis), and instructed level (20 % vs. 80 %). The analysis revealed that performance was poorer (i.e., response variability was higher) while participants were under the influence of cannabis than when they were not *F*(1, 100173.5) = 373.665, *p* < .001, *η*^2^ < 0.01. Furthermore, performance was also poorer as participants were instructed to mind-wander more *F*(1, 47.5) = 4.359, *p* < .05, *η*^2^ = 0.08. There was also a significant interaction between mind-wandering instructions and cannabis use such that the effect of instructions on performance was reduced with cannabis use relative to non-use *F*(1, 100143.3) = 0.326, *p* < .001, *η*^2^ < 0.01.

### 4.3 The relationship between mind-wandering reports and task performance

**Fig. 3:**
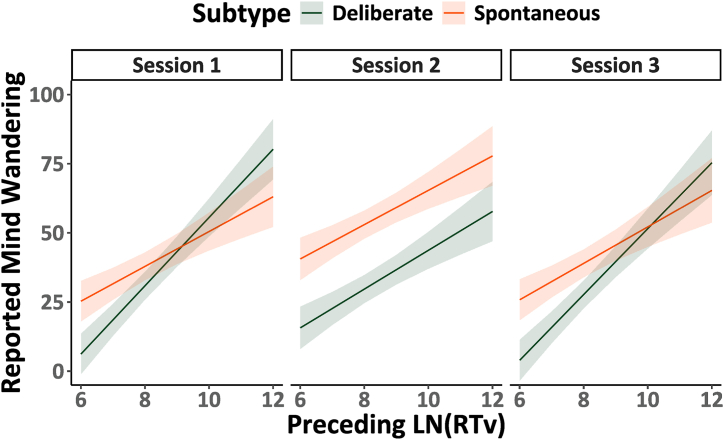
Relationship between mind-wandering reports and preceding task performance. The estimated marginal trends from Model 3.1 for mind-wandering reports are illustrated, faceted by session #, and separated by mind-wandering sub-type.

Finally, we analyzed if the previously reported trend between mind-wandering reports and response variability (RTv) was replicated across sessions in the present study. Model 3.1 in Table 3 examines the relation between mind-wandering reports and RTv in the preceding trials across the experimental sessions (1, 2, 3) while considering potential differences between the mind-wandering subtypes (deliberate, spontaneous). The significant three-way interaction between performance, session and mind-wandering subtype *F*(1, 5092.823) = 3.408, *p* < .05, *η*^2^ < 0.01 is illustrated using marginal trends in Fig. 3.

As may be expected from prior research, across all three sessions greater reports of mind-wandering were associated with poorer performance in the preceding trials, indicated by higher RTv. The relationship between performance and mind-wandering subtypes appears highly similar in Session 1 and Session 3 when participants were not using cannabis. In Session 2 (i.e., when participants were using cannabis) we observed that reports of spontaneous mind-wandering are consistently elevated, and that the slope of the relationship between preceding performance and deliberate mind-wandering reports is reduced. This is in line with our other observations that while under the influence of cannabis participants had a diminished capacity to reduce or increase their deliberate mind-wandering when they were instructed to do so. In summary it appears that the relationship between poorer task performance and increased mind-wandering reports is maintained when participants are under the influence of cannabis.

## 5 Discussion

The present study led to several primary observations. First, relative to the sober state, cannabis use increased both deliberate and spontaneous mind-wandering, with a much larger increase in spontaneous than deliberate mind-wandering. That cannabis inhalation primarily increased uncontrolled (spontaneous) mind-wandering states is consistent with the conclusion that cannabis leads to a loss of attentional control [[5], [6], [7]]. The slight cannabis-related increase in deliberate mind-wandering could be explained in at least two ways. One possibility is the cannabis-related increase in spontaneous mind-wandering creates more situations in which participants deliberately decide to continue spontaneously triggered episodes. Another possibility is cannabis reduces one's motivation to perform the primary task, increasing deliberate shifts of attention from the task. Since we did not measure motivation levels, we are unable to adjudicate between these alternatives.

Second, we found relative to the sober state, cannabis use reduced people's ability to modulate their deliberate mind-wandering levels to match instructed amounts, without noticeably influencing instruction-related modulation of spontaneous mind-wandering. Regarding deliberate mind-wandering, participants under the influence of cannabis were unable to reduce it as effectively when instructed to mind-wander 20 % of the time, and they were also unable to increase it as much when instructed to mind-wander 80 % of the time. This pattern extends prior work by showing that cannabis use impairs the capacity to regulate experiences of mind-wandering. The finding that cannabis impairs control of deliberate states of mind-wandering is consistent both with the notion that cannabis impairs attentional control, and with the assumption that deliberate mind-wandering reflects intentional and controlled processing. That cannabis use does not seem to impair control of spontaneous mind-wandering supports the assumption that these states are driven by distinct regulatory control mechanisms. Thus, the present findings not only inform us about the attentional mechanisms affected by cannabis use, but they also buttress our understanding of spontaneous and deliberate mind-wandering states as involving different underlying attentional mechanisms. The striking dissociation between deliberate and spontaneous mind-wandering is consistent with a growing body of literature showing that these two types of experiences are related differently to a variety of factors including difficulty, motivation, alertness, and working memory capacity [23,25,56].

Third, we consistently showed cannabis use led to increased response time variability during the attention task, which supports prior studies demonstrating a strong link between increases in mind-wandering and poorer performance on attention tasks [[33], [34], [35],^57^]. We replicated prior trends between response variability in the MRT and mind-wandering reports [[33], [34], [35]] demonstrating that response variability continues to serve as a useful objective correlate of mind-wandering even when individuals are under the influence of cannabis. Cannabis use has psychomotor effects (i.e. slower response times, and impaired motor control) that may have contributed to the present finding [6]. However, the interaction whereby the effect of our instructions on performance were reduced when participants were using cannabis suggests performance differences were not solely the result of the psychomotor properties of the drug. Cannabis use impairing both performance and the ability to follow instructions further supports our interpretation that cannabis impairs attentional regulation.

There were several limitations to our experimental design. Specifically, with cannabis self-administration it was not possible to blind participants to their session condition, which raises the concern that task engagement and mind-wandering self-reports may have been influenced by demand characteristics (i.e., response bias). However, there are several trends in the data which present inconvenient challenges to a demand characteristic account. First, one would expect that if participants’ responses were motivated by bias, deliberate mind-wandering reports would be more affected by the administration of cannabis than spontaneous mind-wandering reports, as deliberate mind-wandering is ostensibly under more conscious control; however, in the baseline blocks we found the opposite to be true. Second, during the instructed blocks, we would expect demand characteristics to lead cannabis use to be consistently associated with greater mind-wandering reports; however, we found that when participants were instructed to mind-wander 80 % of the time, their deliberate mind-wandering reports were significantly reduced under the influence of cannabis relative to when they were sober. Third, since participants were not informed that response variability was being used as an index of task performance, we might expect that a cannabis-related response bias ought to influence mind-wandering reports and not response variability, thus reducing the relation between the two measures; however, we found that the relation between mind-wandering reports and response variability was unaffected by cannabis use. In addition to these data driven arguments we would like to note that with psychoactive substances, like cannabis, it is difficult to truly blind users, as their subjective experiences would likely be correlated with the presence or absence of the substance, and possibly its dose.

We were also limited in the precision with which we were able to index the potential physiological impact of THC and CBD because 1) there were variations across individuals in administration technique (e.g., depth of inhalation), 2) there were likely individual differences in drug tolerance (i.e., magnitude of compensatory responses), and 3) our method of measuring THC and CBD in the products was limited by self-report and the accuracy of available product information. This, together with limitations in our sample size precluded us from meaningfully relating THC and CBD concentrations and the observed effects on mind-wandering and performance. In future studies, these relations could be addressed with a much larger sample, better physiological indices of drug bioavailability, and designs that allowed for systematic variations in drug dosage with a given individual so that a drug dose response curve can be estimated.

However, our study also had several strengths. First, in our models we controlled for sleepiness as a potential confounding factor, which had not been examined previously in the existing cannabis and mind-wandering literature [40,41]. This is important because prior studies have established that sleepiness and mind-wandering frequently co-occur [44] and that cannabis use can induce sleepiness [43]. Our results clearly show that cannabis use influences mind-wandering and performance even when sleepiness is statistically controlled. Second, the naturalistic approach we employed, in which participants self-administered cannabis, allowed us to examine the effects of a broad sample of products popular with consumers, many of which had potencies (e.g. 50 % THC) considerably higher than what has previously been administered in laboratory studies [58]. Considering the health and public policy implications associated with the effects of cannabis on cognition, there are considerable benefits to examining the effects of the drug at doses being used by the public. Prior studies of cannabis and mind-wandering involved administering oral doses of 7.5 mg or 15 mg of THC to participants [40,41]; we estimated our participants inhaled on average 83.45 mg of THC. As inhaled cannabis has a bioavailability 2-3x greater than that of orally ingested cannabis [59], this represents a considerable difference in participant THC dosage being examined.

The present findings, namely that cannabis use increases mind-wandering and impairs the regulation of mind-wandering, are aligned with the existing body of literature which shows that cannabis use impairs performance on attention-related tasks. However, despite this evidence, there is a popular perception amongst cannabis users that the drug can promote focus and productivity [60]. Future studies may seek to explore why this misalignment between cannabis user's perceptions and objective evaluations of performance on cognitive tasks occurs. One potential avenue involves the influence of cannabis on meta-awareness of mind-wandering, which refers to individuals' awareness of whether they are mind-wandering as it is occurring [61]. Considering that cannabis use has been linked to impairments of other meta-cognitive abilities such as error-awareness [62], individuals using cannabis to focus might perceive that they are more attentive due to the drug impairing their meta-awareness of mind-wandering, thus producing a false sense of overconfidence. We were unable to address this issue in the present study because we used a probe-caught approach for detecting mind-wandering, which is effective at capturing mind-wandering regardless of whether an individual has meta-awareness of the episode as it unfolds [61]. Perhaps a better approach for this purpose would be to use the “self-caught method”, whereby participants are not probed, but are asked simply to report whenever they find their mind has wandered from the task [63]. If meta-awareness is impaired by cannabis use, then there ought to be fewer self-caught episodes of mind wandering when cannabis is used than when it is not. Studies along these lines will yield further insights into the influence of cannabis on inattention and attentional regulation.

## CRediT authorship contribution statement

**Adrian Berk Safati:** Writing – review & editing, Writing – original draft, Visualization, Software, Project administration, Methodology, Investigation, Formal analysis, Conceptualization. **Wisam Almohamad Alkheder:** Writing – original draft, Methodology, Investigation. **Cassandra Justine Lowe:** Writing – review & editing, Formal analysis. **Daniel Smilek:** Writing – review & editing, Writing – original draft, Supervision, Methodology, Funding acquisition, Conceptualization.

## Data availability

Study materials, data, and analysis scripts are available at https://osf.io/43ur6/?view_only=f68a5f08dad448a89a8b172e057321cb.

## Ethics

This research adhered to the principles of the Declaration of Helsinki and was approved by the Office of Research Ethics at the University of Waterloo (approval number 45606), informed consent was obtained from all subjects.

## Funding

This research was supported by a 10.13039/501100000038NSERC (10.13039/501100000038Natural Sciences and Engineering Research Council) Discovery Grant awarded to Daniel Smilek (RGPIN-2019-04071) and a 10.13039/501100000038NSERC Canada Graduate Scholarships – Doctoral Scholarship award to Adrian Safati.

## Declaration of competing interest

The authors declare that they have no known competing financial interests or personal relationships that could have appeared to influence the work reported in this paper.
