# Supplementary material for: The effects of cannabis on mind-wandering
Source: Heliyon. 2025 Feb 21;11(4):e42911. doi: 10.1016/j.heliyon.2025.e42911 (PMC11909433; doi:10.1016/j.heliyon.2025.e42911)
Supplement: Multimedia component 2 [file mmc2.docx]

# Supplementary Materials

These supplementary materials contain three sections.

***1. Analysis of Mind-Wandering and Task Performance by Session Number****.*

**2. Analysis of Sleepiness as a Function of Session Number and Cannabis Use**.

**3. Highlights of Sample Characteristics**.

**1. Analysis of Mind-Wandering and Task Performance by Session Number**

In the main paper the results are presented as a function of cannabis use with the data from Session 1 and Session 3 (when participants were not using cannabis) combined and contrasted with data from Session 2 (when participants were using cannabis). Here the analysis and comparisons examine all three sessions separately. The models presented herein follow a highly similar template to those presented in the main paper with the Models in Table S1 and Table S2 corresponding to their counterparts in Table 1 and Table 2 with the exception that the results are separated as a function of session number, instead of session cannabis use. Models S1.1-S1.2 examine the data from the ‘baseline’ (non-instructed) block and Models S2.1-S2.2 examine the data from the instructed blocks. In all of the models average sleepiness scores from the KSS administered at the beginning and end of each block were included in the fixed effects to account for sleepiness as a potential confounding factor. In Models S1.1 and S2.1 we included separate intercepts for spontaneous and deliberate mind-wandering reports as these subtypes are distinct constructs. While Model S1.2 included random slopes for the effect of instructions to better account for individual differences in the response to the instructions to mind-wander 20% or 80% of the time, Model S2.2 only included random intercepts to promote model convergence.

## The Influence of Cannabis During Baseline

We began by analyzing data from the baseline block. Fig. S1a-b show mind-wandering responses in the baseline block as a function of type of report (Deliberate, Spontaneous) and session number (1, 2, 3). Analyses of the data (Model S1.1, Table S1) revealed highly similar findings to those presented in the main paper. First, there was a main effect of mind-wandering type, such that overall people reported more spontaneous than deliberate mind-wandering. Second, there was a main effect of session, whereby overall mind-wandering was greater when participants were under the influence of cannabis (during Session 2) then when they were sober (in Sessions 1 & 3). Third, the effect of cannabis use was much more prominent for spontaneous than for deliberate mind-wandering. Fig. S1c includes the MRT RT variability in the baseline block as a function of cannabis use. Analyses of these data (Model S1.2; Table S1) revealed a main effect of session whereby performance was poorer (RT variance was higher) right after participants smoked cannabis (in Session 2) then sessions when they were sober (Sessions 1 & 3). The significant improvement in task performance observed between Session 1 and Session 3, may be indicative of a practise related improvement across the sober sessions.


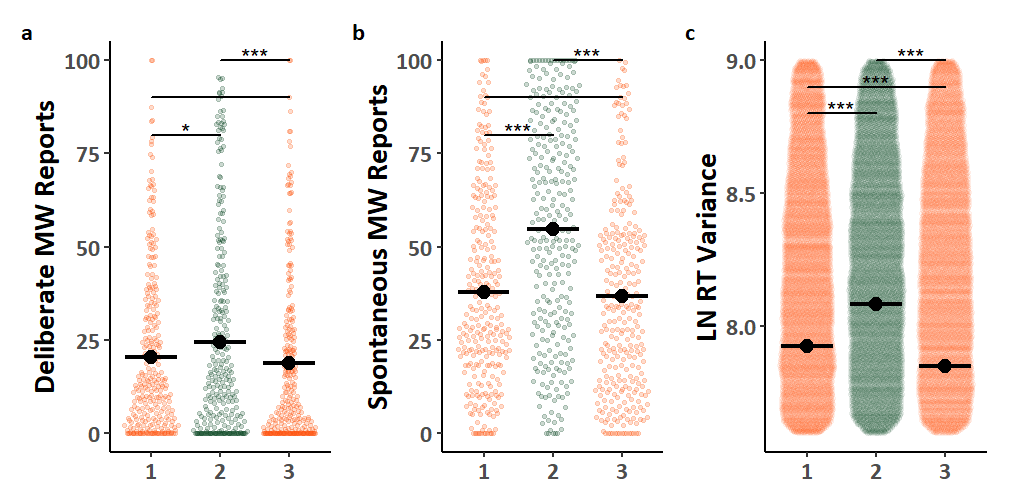


Fig. S1. Mind-wandering reports and MRT performance during baseline blocks. The coloured points illustrate raw participant responses, with the y-axis for Panel c) restricted to better highlight mean differences. The estimated marginal means from Model S1.1 for Panels a) & b), Model S1.2 for Panel c) are presented in black. Pairwise comparisons with significance scores are shown at the top, Tukey’s HSD was used to adjust for multiple comparisons. The rhythmic response time variability scores presented in Panel c) was transformed by applying a natural logarithm function. (* = *p* < 0.05, ** = *p* < 0.01, *** = *p* < .001)

## Table S1

ANOVA results for Linear Mixed Effects Models Examining Baseline Blocks.

| **Model** | **Parameter** | ***SS*** | ***MS*** | ***df_Num_*** | ***df_Den_*** | ***F*** | ***p*** |
| --- | --- | --- | --- | --- | --- | --- | --- |
| **Model S1.1** | Model Specification: MW ~ Instruction*Session*Type + KSS + (1 \| ID:Type)  Model Data: Baseline Blocks, Observations = 1728  Performance: *R*^2^_Marginal_ = 0.212, *R*^2^_Conditional_ = 0.608 | | | | | | |
|  | Session | 44960.57 | 22480.28 | 2 | 1643.7 | 69.158 | <0.000 |
|  | Type | 10774.77 | 10774.77 | 1 | 94.0 | 33.147 | <0.000 |
|  | KSS | 9354.55 | 9354.55 | 1 | 1720.3 | 28.778 | <0.000 |
|  | Session:Type | 15063.22 | 7531.61 | 2 | 1627.1 | 23.170 | <0.000 |
|  |  |  |  |  |  |  |  |
| **Model S1.2** | Model Specification: LN_RTv ~ Session + KSS + (1 \| ID)  Model Data: All Blocks, Observations = 50179  Performance: *R*^2^_Marginal_ = 0.006, *R*^2^_Conditional_ = 0.211 | | | | | | |
|  | Session | 407.67 | 203.83 | 2 | 50159.3 | 171.599 | <0.000 |
|  | KSS | 0.1 | 0.1 | 1 | 49537.5 | 0.08 | 0.777 |

Predictor significance was assessed using the Satterthwaite approximation for degrees of freedom.

## The Influence of Cannabis on Directed Mind-Wandering

Next, we analyzed data from the two mind-wandering instruction blocks (20% and 80% instructed mind-wandering). Mind-wandering reports in the instructed blocks are depicted in Fig. 2a-b as a function of mind-wandering type (Deliberate, Spontaneous), session (1, 2, 3) and instructions (20%, 80%). The analysis (Model S2.1, Table S2) again revealed highly similar findings to those presented in the main paper. There was a significant increase in mind-wandering from the 20% to 80% instruction blocks (main effect of instructions), indicating that instructions were effective, as well as a larger impact of instructions on deliberate than spontaneous mind-wandering (the instruction by type interaction). Decomposing the three-way interaction, we analyzed the deliberate and spontaneous mind-wandering reports separately. Analysis of deliberate mind-wandering reports (see Model S2.1a; Table S2) revealed a significant instruction by session interaction. This interaction was driven by a smaller effect of instructions when participants were under the influence of cannabis (Session 2), than when they were not (Sessions 1 & 3). In contrast, analysis of spontaneous mind-wandering reports (Model S2.1b; Table S2) showed that cannabis use, and the mind-wandering instructions had independent effects on these reports, as the instruction by session interaction did not reach significance.

The MRT RT variability data from the instructed blocks are shown in Fig. S2c. They were analyzed (see Model S2.2; Table S2) as a function of session (1, 2, 3), and instructed level (20% vs. 80%). The analysis revealed that performance was poorer (i.e., response variability was higher) while participants were under the influence of cannabis (Session 2) than when they were not (Sessions 1 and 3). Furthermore, performance was also poorer as participants were instructed to mind-wander more. There was also a significant interaction between mind-wandering instructions and sessions.


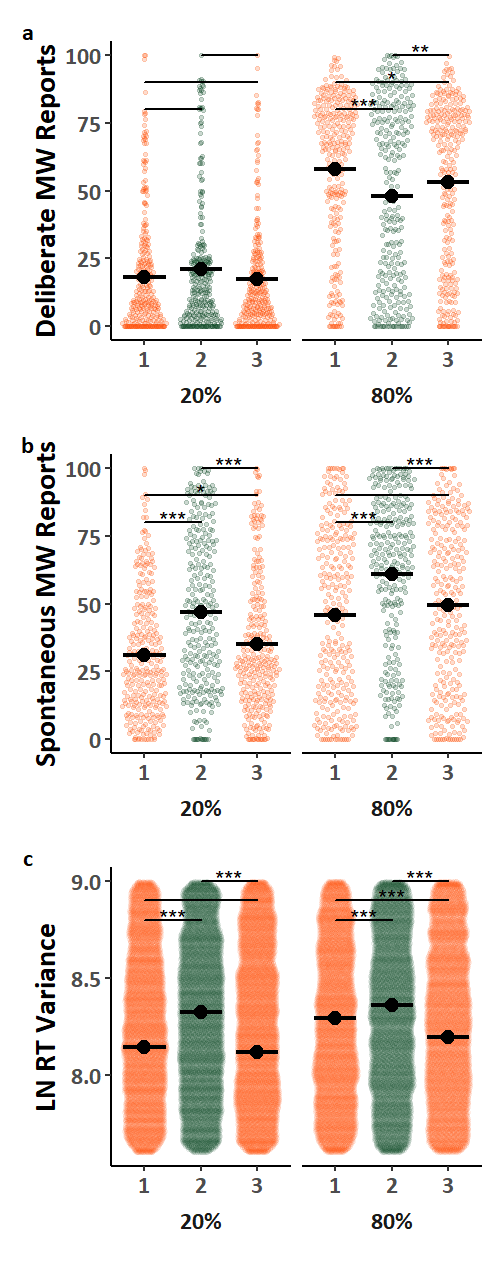


Fig. S2. Mind-wandering reports and MRT performance during directed blocks. The coloured points illustrate raw participant responses, with the y-axis for Panel c) restricted to better highlight mean differences. The estimated marginal means from Model S2.1 for Panels a) and b), and Model S2.2 for Panel c), are presented in black. Pairwise comparisons with significance scores are shown at the top, Tukey’s HSD was used to adjust for multiple comparisons. The rhythmic response time variability scores presented in Panel c) were transformed by applying a natural logarithm function. (* = *p* < 0.05, ** = *p* < 0.01, *** = *p* < .001)

## Table S2

ANOVA results for Linear Mixed Effects Models Examining Instructed Blocks.

| **Model** | | **Parameter** | | ***SS*** | | ***MS*** | | ***df_Num_*** | | ***df_Den_*** | | ***F*** | ***p*** | |
| --- | --- | --- | --- | --- | --- | --- | --- | --- | --- | --- | --- | --- | --- | --- |
| **Model S2.1** | | Model Specification: MW ~ Instruction*Session*Type + KSS + (Instruction \| ID:Type)  Model Data: Instructed Blocks, Observations = 3454  Performance: *R*^2^_Marginal_ = 0.237, *R*^2^_Conditional_ = 0.582 | | | | | | | | | | | | |
|  | | Instruction | | 82619.71 | | 82619.71 | | 1 | | 93.8 | | 197.46 | <0.000 | |
|  | | Session | | 24555.79 | | 12277.9 | | 2 | | 3278.7 | | 29.344 | <0.000 | |
|  | | Type | | 2651.81 | | 2651.81 | | 1 | | 93.7 | | 6.338 | 0.014 | |
|  | | KSS | | 21831.16 | | 21831.16 | | 1 | | 3071.1 | | 52.176 | <0.000 | |
|  | | Instruction:Session | | 7046.51 | | 3523.25 | | 2 | | 3253.2 | | 8.421 | <0.000 | |
|  | | Instruction:Type | | 13839.37 | | 13839.37 | | 1 | | 93.8 | | 33.076 | <0.000 | |
|  | | Session:Type | | 53678.03 | | 26839.02 | | 2 | | 3252.3 | | 64.145 | <0.000 | |
|  | | Instruction:Session:Type | | 5204.48 | | 2602.24 | | 2 | | 3252.2 | | 6.219 | 0.002 | |
|  | |  | |  | |  | |  | |  | |  |  | |
| **Model S2.1a** | | Model Specification: Deliberate ~ Instruction*Session + KSS + (Instruction \| ID)  Model Data: Instructed Blocks, Observations = 1727  Performance: *R*^2^_Marginal_ = 0.295, *R*^2^_Conditional_ = 0.598 | | | | | | | | | | | | |
|  | | Instruction | | 67829.15 | | 67829.15 | | 1 | | 47 | | 164.095 | <0.000 | |
|  | | Session | | 5969.85 | | 2984.93 | | 2 | | 1641.3 | | 7.221 | 0.001 | |
|  | | KSS | | 193.72 | | 193.72 | | 1 | | 1349.4 | | 0.469 | 0.494 | |
|  | | Instruction:Session | | 11195.99 | | 5598 | | 2 | | 1626.5 | | 13.543 | <0.000 | |
|  | |  | |  | |  | |  | |  | |  |  | |
| **Model S2.1b** | | Model Specification: Spontaneous ~ Instruction*Session + KSS + (Instruction \| ID)  Model Data: Instructed Blocks, Observations = 1727  Performance: *R*^2^_Marginal_ = 0.170, *R*^2^_Conditional_ = 0.552 | | | | | | | | | | | | |
|  | | Instruction | | 18222.37 | | 18222.37 | | 1 | | 46.7 | | 43.958 | <0.000 | |
|  | | Session | | 71907.3 | | 35953.65 | | 2 | | 1637.8 | | 86.731 | <0.000 | |
|  | | KSS | | 38206.34 | | 38206.34 | | 1 | | 1627 | | 92.165 | <0.000 | |
|  | | Instruction:Session | | 168.76 | | 84.38 | | 2 | | 1626 | | 0.204 | 0.816 | |
|  | |  | |  | |  | |  | |  | |  |  | |
| **Model S2.2** | Model Specification: LN_RTv ~ Instruction*Session + KSS + (1 \| ID)  Model Data: All Blocks, Observations = 100240  Performance: *R*^2^_Marginal_ = 0.014, *R*^2^_Conditional_ = 0.258 | | | | | | | | | | | | | |
|  | Instruction | | 194.36 | | 194.36 | | 1 | | 100186.2 | | 145.612 | | | <0.000 |
|  | Session | | 558.02 | | 279.01 | | 2 | | 100201.1 | | 209.036 | | | <0.000 |
|  | KSS | | 575.65 | | 575.65 | | 1 | | 99577.1 | | 431.28 | | | <0.000 |
|  | Instruction:Session | | 54.29 | | 27.15 | | 2 | | 100186.5 | | 20.337 | | | <0.000 |

Predictor significance was assessed using the Satterthwaite approximation for degrees of freedom.

## 2. Analysis of Sleepiness as a Function of Session Number and Cannabis Use

In the analysis presented in the main paper we attempted to control for sleepiness based on prior reports that cannabis can promote sleepiness [1] and because sleepiness and mind-wandering have correlated but dissociable influences on task performance [2]. By asking participants to rate their sleepiness on the Karolinska Sleepiness Scale (KSS) [3] at the start and end of each block we could use the average of the reports for a given block to control for sleepiness as a potential confounding factor. In these supplemental materials we present sleepiness modelled as the dependent variable as a function of both cannabis use (as shown in the main paper) and as a function of session number (as shown in the supplementary materials).

Following the template of prior models, we examine the baseline and instructed blocks separately; the primary difference herein is the analysis of block averaged sleepiness reports from the KSS as the dependent outcome rather than as a control variable. Models S3.1 and S3.2 examine sleepiness reports in the baseline blocks and Models S4.1 and S4.2 examine sleepiness reports in the instructed blocks, with random slopes for differences in the level of instruction. While Models S3.1 and S4.1 examine the reports as a function of session number as shown earlier in the supplementary materials, Models S3.2 and S4.2 examine the reports as a function of cannabis use as shown in the main paper.

## The Influence of Cannabis During Baseline on Sleepiness Reports

Fig. S3 shows sleepiness reports in the baseline block as a function of session (1, 2, 3) in Fig. S3a or as a function of cannabis use in Fig. S3b. Recall that participants were sober in Sessions 1 and 3 and under the influence of cannabis in Session 2. Analyses by session (see Models S3.1; Table S3) reveals a main effect of session such that sleepiness scores were greater in Session 1 than Session 3, and in Session 2 relative to Session 3. Analysis by cannabis use (Models S3.2, Table S3) shows that sleepiness scores were greater when participants were under the influence of cannabis relative to when they were not using cannabis.

**
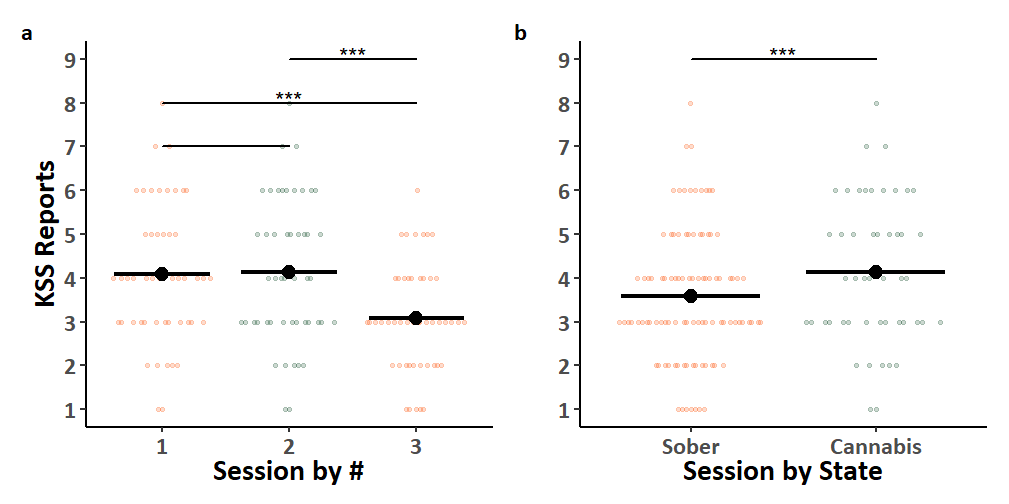
**

Fig. S3. KSS scores during baseline blocks. The coloured points illustrate the block averages of participant’s responses. While Panel a) presents the three experimental sessions separately, Panel b) combines Sessions 1 & 3 (when participants were sober). The estimated marginal means from Model S3.1 for Panels a), and Model S3.2 for Panel b) are presented in black. Pairwise comparisons with significance scores are shown at the top, Tukey’s HSD was used to adjust for multiple comparisons. (* = *p* < 0.05, ** = *p* < 0.01, *** = *p* < .001)

## Table S3

ANOVA Tables for the Linear Mixed Effects Models Examining KSS Reports.

| **Model** | **Parameter** | ***SS*** | ***MS*** | ***df_Num_*** | ***df_Den_*** | ***F*** | ***p*** |
| --- | --- | --- | --- | --- | --- | --- | --- |
| **Model S3.1** | Model Specification: KSS ~ Session + (1 \| ID)  Model Data: Baseline Blocks, Observations = 147 Performance: *R*^2^_Marginal_ = 0.096, *R*^2^_Conditional_ = 0.383 | | | | | | |
|  | Session | 34.79 | 17.39 | 2 | 96 | 11.343 | <0.000 |
|  |  |  |  |  |  |  |  |
| **Model S3.2** | Model Specification: KSS ~ Cannabis + (1 \| ID)  Model Data: Baseline Blocks, Observations = 147  Performance: *R*^2^_Marginal_ = 0.028, *R*^2^_Conditional_ = 0.284 | | | | | | |
|  | Cannabis | 10.29 | 10.29 | 1 | 97 | 5.812 | 0.018 |

Predictor significance was assessed using the Satterthwaite approximation for degrees of freedom.

### The Influence of Cannabis and Instructions on Sleepiness Reports

Fig. S4 shows sleepiness reports in the instructed blocks as a function of session (1, 2, 3) and instructions (20%, 80%) in Fig. S4a, or as a function of cannabis use and instructions (20%, 80%) in Fig. S4b. Analyses by session (Model S4.1, Table S4) revealed a significant effect of session replicating prior findings, but no interaction between session and instructions. As in the baseline block sleepiness scores were greater in Session 1 than Session 3, and in Session 2 relative to Session 3. Analysis by cannabis use (Model S4.2, Table S4), again replicated prior findings with no interaction between cannabis use and instructions. As in the baselines block sleepiness scores were greater when participants were using cannabis. Reports of sleepiness appear to be unaffected by instructions to mind-wander different amounts with no significant main effect of instructions on sleepiness reports in Models S4.1 or S4.2 (Table S4), and no significant interaction between instructions and session or cannabis use.

The significant differences in sleepiness scores as a function of both session number and cannabis use supports our consideration of sleepiness as a potential confound in the prior models presented. It is not clear why sleepiness reports were lower in Session 3 compared to Session 1, but this finding is in line with our prior observation that task performance was significantly improved in Session 3 compared to Session 1.

*
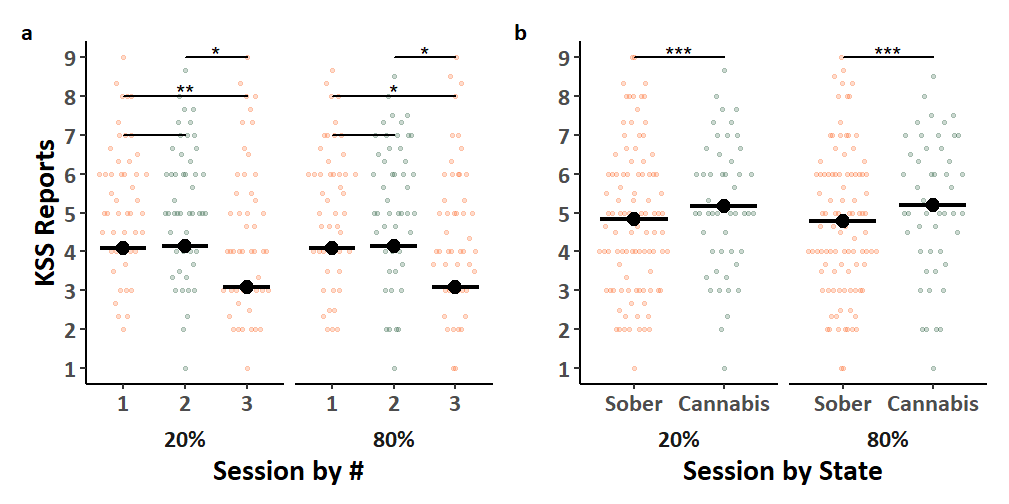
*

Fig. S4. KSS scores during directed blocks. The coloured points illustrate the block averages of participant’s responses. While Panel a) presents the three experimental sessions separately, Panel b) combines Sessions 1 & 3 (when participants were sober). The estimated marginal means from Model S4.1 for Panels a), and Model S4.2 for Panel b) are presented in black. Pairwise comparisons with significance scores are shown at the top, Tukey’s HSD was used to adjust for multiple comparisons. (* = *p* < 0.05, ** = *p* < 0.01, *** = *p* < .001)

## Table S4

ANOVA Tables for the Linear Mixed Effects Models Examining KSS Reports.

| **Model** | **Parameter** | ***SS*** | ***MS*** | ***df_Num_*** | ***df_Den_*** | ***F*** | ***p*** |
| --- | --- | --- | --- | --- | --- | --- | --- |
| **Model S4.1** | Model Specification: KSS ~ Instruction*Session + (Instruction \| ID)  Model Data: Instructed Blocks, Observations = 294  Performance: *R*^2^_Marginal_ = 0.036, *R*^2^_Conditional_ = 0.534 | | | | | | |
|  | Instruction | 0.07 | 0.07 | 1 | 239.1 | 0.044 | 0.835 |
|  | Session | 34.46 | 17.23 | 2 | 240 | 10.910 | <0.000 |
|  | Instruction:Session | 0.80 | 0.40 | 2 | 240 | 0.252 | 0.777 |
|  |  |  |  |  |  |  |  |
| **Model S4.2** | Model Specification: KSS ~ Instruction*Session + (Instruction \| ID)  Instructed Blocks, Observations = 294  Performance: *R*^2^_Marginal_ = 0.009, *R*^2^_Conditional_ = 0.505 | | | | | | |
|  | Instruction | 0.02 | 0.02 | 1 | 241.3 | 0.010 | 0.920 |
|  | Cannabis | 9.10 | 9.10 | 1 | 242 | 5.439 | 0.021 |
|  | Instruction:Cannabis | 0.12 | 0.12 | 1 | 242 | 0.073 | 0.787 |

Predictor significance was assessed using the Satterthwaite approximation for degrees of freedom.

## 4. Highlights of Sample Characteristics

To better characterize our sample population we asked participants to complete a series of questionnaires including basic demographic information (age, sex, race), the Daily Sessions, Frequency, Age of Onset, and Quantity of Cannabis Use Inventory (DFAQ-CU) [4], and the Marijuana Motives Questionnaire (MMQ) [5]. Additionally with their planned use of cannabis preceding Session 2 participants were asked to upload photos of the product label, the joint pre- and post-consumption, to report how “high” they felt, whether they shared the joint with others, and if so, what fraction they consumed, and to what extent they felt they “inhaled” while smoking. The full contents of these questionnaires, along with information about the cannabis products used by participants are available on our OSF repository <https://osf.io/43ur6/?view_only=f68a5f08dad448a89a8b172e057321cb>. Herein we provide a summary and highlights of the collected information.

### Demographics

The demographic results (Table S5) reveal a young *M* = 23.13 ± 5.57 (19-53) but diverse sample with a variety of racial identities, and a balanced representation of sexes (Female = 24, Male = 23, Unreported = 1).

### Dosage Survey

The results from the dosage survey (Table S5) indicated that the concentrations of THC and CBD in the products used by participants ranged from THC *M* = 252.71 mg/g ± 62.29 (88 – 500), CBD *M* = 3.32 mg/g ± 15.05 (0 – 104). As indicated in the main paper we found that participants self-administered average doses of THC *M* = 83.45 mg ± 47.24 (3.01 – 246.12), and CBD *M* = 0.52 mg ± 1.31 (0 – 8.50). In our sample 47 of 48 participants analysed were using high THC cannabis products, which meant that the doses of CBD people used were limited (i.e. with 45 estimated to be <1mg).

While product labels suggest that participants self-selected a variety of plant strains (Indica = 23, Sativa = 15, Hybrid = 10), these categorizations should be considered critically as the labelling of a products e.g. as “Sativa Dominant”, or “Indica Dominant Hybrid” makes it difficult to ascertain the extent to which hybridization has occurred or whether these categorizations have meaningful implications for psychoactive constituents of the product.

### Marijuana Motives Questionnaire (MMQ)

The MMQ is a 25-item scale scoring 5 motivating factors for cannabis use (Enhancement, Conformity, Expansion, Coping, and Social) on a scale from 1 – “Never/Almost Never” to 4 – “Almost Always/ Always” [5]. The MMQ results (Table S5) indicated the highest scoring motivation for cannabis use in our sample was “Enhancement”, whereas “Conformity” was the lowest scoring factor.

### Daily Sessions, Frequency, Age of Onset, and Quantity of Cannabis Use Inventory (DFAQ-CU)

The DFAQ-CU is a 39-item inventory with 24 core items designed to measure frequency, age of onset, and quantity of cannabis use [4]. Two items i.e., “have you ever smoked cannabis”, and “how high are you right now” were redundant with our study design and excluded. The DFAQ-CU results (Table S5) indicate an average onset of cannabis use in our sample 17.36 ± 2.32 (14 – 27), that is below the legal purchasing age in the province of 19, this is in line with prior uses of the DFAQ-CU [4].

One unexpected observation from the DFAQ-CU results was that several participants reported smoking on average less or more than the 2-8 times per month outlined in our recruitment materials. We had sought to recruit regular users as participants so we could reliably schedule sessions around their planned use (or non-use) of cannabis. We do not believe that recruited individuals with different average frequency of use than intended had a substantial impact on the study findings.

## Table S5

*Demographics, Cannabis Dose, Motivations, and Habits*

| Category | *n* or *Mean* ± *SD* (Range) |
| --- | --- |
| Demographics |  |
|  |  |
| Sex | Female = 24, Male = 23, Unreported = 1 |
| Age (Years) | 23.13 ± 5.57 (19-53) |
|  |  |
| Race: |  |
| Asian | 7 |
| Black/African | 1 |
| Middle Eastern | 3 |
| South Asian | 15 |
| White/Caucasian | 13 |
| Mixed | 7 |
| Other | 1 |
|  |  |
| Dosage (Dosage Survey) |  |
|  |  |
| Product THC (mg/g) | 252.71 ± 62.29 (88 – 500) |
| Product CBD (mg/g) | 3.32 ± 15.05 (0 – 104) |
| THC Dose Estimate (mg) | 83.45 ± 47.24 (3.01 – 246.12) |
| CBD Dose Estimate (mg) | 0.52 ± 1.31 (0 – 8.50) |
| Amount Inhaled (0-100) | 70.69 ± 20.30 (20 – 100) |
| Subjective High (0-100) | 62.19 ± 20.13 (10 – 100) |
|  |  |
| Plant Type: |  |
| Indica | 23 |
| Sativa | 15 |
| Hybrid | 10 |
|  |  |
| Motives (MMQ) |  |
|  |  |
| Enhancement (1-4) | 3.08 ± 0.56 (1.8 – 4) |
| Conformity (1-4) | 1.21 ± 0.34 (1 – 2.4) |
| Expansion (1-4) | 2.00 ± 0.79 (1 – 4) |
| Coping (1-4) | 2.20 ± 0.71 (1 – 3.75) |
| Social (1-4) | 2.00 ± 0.58 (1 – 3.4) |
|  |  |
| Habits (DFAQ-CU) |  |
|  |  |
| Age First Tried | 17.36 ± 2.32 (14 – 27) |
|  |  |
| Average Frequency of Use: |  |
| Once every 2 months | 2 |
| Once a month | 2 |
| 2-3 times a month | 9 |
| Once a week | 8 |
| Twice a week | 11 |
| 3-4 times a week | 10 |
| 5-6 times a week | 2 |
| Once a day | 2 |
| More than once a day | 1 |
|  |  |
| Average Session Use (g)^*^ | 0.378 ± 0.25 (0-1) |
|  |  |
| Primary Form of Use: |  |
| Marijuana | 40 |
| Concentrates | 2 |
| Edibles | 5 |

^*^There was a high incidence of missing responses for this item.
*Note*. There were additional items from the DFAQ-CU that are not reported in the present table, this data is available on our OSF repository.

# References

[1] R. Velzeboer, A. Malas, P. Boerkoel, K. Cullen, M. Hawkins, J. Roesler, W.W.-K. Lai, Cannabis dosing and administration for sleep: a systematic review, Sleep 45 (2022) zsac218.

[2] D. Stawarczyk, A. D’Argembeau, Conjoint influence of mind-wandering and sleepiness on task performance, J Exp Psychol Hum Percept Perform 42 (2016) 1587–1600. https://doi.org/10.1037/xhp0000254.

[3] T. Åkerstedt, M. Gillberg, Subjective and objective sleepiness in the active individual, International Journal of Neuroscience 52 (1990) 29–37.

[4] C. Cuttler, A. Spradlin, Measuring cannabis consumption: Psychometric properties of the daily sessions, frequency, age of onset, and quantity of cannabis use inventory (DFAQ-CU), PloS One 12 (2017) e0178194.

[5] J. Simons, C.J. Correia, K.B. Carey, B.E. Borsari, Validating a five-factor marijuana motives measure: Relations with use, problems, and alcohol motives., Journal of Counseling Psychology 45 (1998) 265.
